# Multiple sclerosis disease activity, a multi-biomarker score of disease activity and response to treatment in multiple sclerosis

**DOI:** 10.3389/fimmu.2024.1338585

**Published:** 2024-06-27

**Authors:** Alexandru Tatomir, Freidrich Anselmo, Dallas Boodhoo, Hegang Chen, Armugam P. Mekala, Vinh Nguyen, Jacob Cuevas, Violeta Rus, Horea Rus

**Affiliations:** ^1^ Department of Neurology, University of Maryland, School of Medicine, Baltimore, MD, United States; ^2^ Neurology Department, Baltimore Veterans Administration Hospital, Baltimore, MD, United States; ^3^ Department of Epidemiology and Public Health, University of Maryland, School of Medicine, Baltimore, MD, United States; ^4^ Department of Medicine, Division of Rheumatology and Clinical Immunology, University of Maryland, School of Medicine, Baltimore, MD, United States

**Keywords:** multiple sclerosis, RGC-32, SIRT1, JNK1, biomarkers, peripheral blood mononuclear cells, glatiramer acetate

## Abstract

Regular assessment of disease activity in relapsing-remitting multiple sclerosis (RRMS) is required to optimize clinical outcomes. Biomarkers can be a valuable tool for measuring disease activity in multiple sclerosis (MS) if they reflect the pathological processes underlying MS pathogenicity. In this pilot study, we combined multiple biomarkers previously analyzed in RRMS patients into an MS disease activity (MSDA) score to evaluate their ability to predict relapses and treatment response to glatiramer acetate (GA). Response Gene to Complement 32 (RGC-32), FasL, IL-21, SIRT1, phosphorylated SIRT1 (p-SIRT1), and JNK1 p54 levels were used to generate cut-off values for each biomarker. Any value below the cutoff for RGC-32, FasL SIRT1, or p-SIRT1 or above the cutoff for IL-21 or JNK1 p54 was given a +1 value, indicating relapse or lack of response to GA. Any value above the cutoff value for RGC-32, FasL, SIRT1, p-SIRT1 or below that for IL-21 or JNK1 p54 was given a -1 value, indicating clinical stability or response to GA. An MSDA score above +1 indicated a relapse or lack of response to treatment. An MSDA score below -1 indicated clinical stability or response to treatment. Our results showed that the MSDA scores generated using either four or six biomarkers had a higher sensitivity and specificity and significantly correlated with the expanded disability status scale. Although these results suggest that the MSDA test can be useful for monitoring therapeutic response to biologic agents and assessing clinically challenging situations, the present findings need to be confirmed in larger studies.

## Introduction

Multiple sclerosis (MS) is an immune-mediated chronic inflammatory disease of the central nervous system (CNS) in which myelin sheaths and oligodendrocytes (OLG) are selectively destroyed, resulting in demyelination and, as the disease progresses, axonal death and ultimately neurodegeneration (1, 2). MS affects mainly young adults, and consequently it has a huge socio-economic impact, being the most frequent cause of nontraumatic neurological disability among young adults in the Western Hemisphere (3). As many as 85% of patients have a relapsing-remitting course (RRMS), a major MS subtype characterized by episodes of neurological symptoms (relapses) followed by partial or complete resolution of symptoms (remission) (3, 4).

Although the etiology of MS remains elusive, recent advances in understanding its pathogenesis have led to the development of effective disease-modifying therapies (DMT) that are able to slow the evolution of the disease (5, 6). These therapies are generally most effective in dampening the inflammatory attacks, since they target key immunological mechanisms responsible for the acute phases of MS. Broadly speaking, these acute events center around multiple cellular effectors, including autoreactive CD4^+^ T cells, cytotoxic CD8^+^ T cells, B cells, macrophages, microglia, and astrocytes, all of which act together to create a pathogenic molecular environment composed primarily of the complement system, pro-inflammatory cytokines, chemokines, free radicals, and other agents that can exert potentially damaging effects on the CNS (7–9). However, as the disease progresses to the chronic phase, these mechanisms eventually become ineffective, perhaps because at this point the pathogenesis shifts toward permanent neurodegeneration (10, 11).

During relapses, it is generally accepted that autoreactive CD4^+^ T cells are activated in the periphery and differentiate toward two main subpopulations with pathogenic potential, namely Th1 and Th17 cells, which can pass the blood-brain barrier and settle into the CNS, where they promote an inflammatory environment together with resident cells such as microglia and astrocytes (12, 13). Since quantitative and regular assessment of disease activity in MS is required to understand if treatment targets are achieved and to optimize clinical outcomes, there is a critical need to identify reliable biomarkers to predict MS relapse and response to therapy (14). The peripheral phase of MS is an important stage at which to identify potential blood-based markers of RRMS, particularly those that identify suppression of the signaling molecules that are specifically involved in T-cell activation.

We have previously demonstrated that several peripheral blood mononuclear cells (PBMC)-based molecules can serve as putative biomarkers in detecting relapses and response to glatiramer acetate (GA) therapy, one of the first and most widely used DMT. Our previous studies have shown that the mRNA levels of Response Gene to Complement 32 (RGC-32), sirtuin 1 (SIRT1), Fas ligand (FasL), and interleukin 21 (IL-21), as well as the protein levels of phosphorylated SIRT1 (p-SIRT1) and c-Jun N-terminal kinases (JNK1) p54 show a good predictability in detecting relapses and response to GA therapy (15–19). Specifically, we have found that the transcript levels of RGC-32, SIRT1, and FasL, as well as the protein levels of p-SIRT1, are significantly reduced in RRMS patients during a relapse and in patients not responding to GA therapy, when compared to RRMS-stable patients and responders to GA, respectively. On the other hand, our studies have also found that the levels of IL-21 mRNA and JNK1 p54 protein are increased during relapses and in non-responders to GA therapy, respectively.

Extensive *in vitro* experiments have shown that these molecules are important players in T-cell signaling, activation, survival, and differentiation (19–22), and, therefore, the variability of their expression (as determined by the patients` clinical status) correlates well with the various pro-inflammatory as well as with the regulatory cellular and molecular processes taking place during inflammatory attacks or periods of recovery, respectively.

In this pilot study, our goal was to enhance the predictive power of the abovementioned biomarkers by combining them into a MS disease activity (MSDA) score, either using a combination of six (RGC-32, IL-21, FasL, JNK1 p54, SIRT1, p-SIRT1) or four biomarkers (RGC-32, IL-21, FasL, JNK1 p54), in order to better detect relapses and response to GA therapy. Our preliminary results show that the MSDA scores for both four and six biomarkers had a better sensitivity and specificity than most of the six biomarkers taken individually, and they correlated significantly with the expanded disability status scale (EDSS) results, suggesting that the MSDA score could become one of the most useful peripheral blood-based biomarkers for following RRMS patients.

## Materials and methods

### Patient enrollment

Fifteen RRMS patients were enrolled from the University of Maryland Multiple Sclerosis Center. This cohort consisted of nine females (60% of the total patients) and six males (40% of the total patients), with a mean age of 40 (range, 22–60). The inclusion criteria for these patients were: (i) age 18 to 65 years; (ii) fulfillment of the McDonald criteria for definitive MS (23); (iii) a relapsing-remitting course; (iv) newly diagnosed MS, or MS not treated with interferon-β or GA for the last 3 months prior to study entry; (v) no exacerbations in the last 4 weeks prior to study entry; (vi) no intravenous or oral steroids for the last 4 weeks prior to study enrollment; (vii) no treatment with any of the following DMT: anti-CD20 monoclonal antibodies, natalizumab, fingolimod, mitoxantrone, cyclophosphamide, alemtuzumab, teriflunomide, or any investigational drug during the past year prior to study entry; and (viii) a disability score of 0–5.5, as defined by the EDSS (24). Exclusion criteria for MS patients were: (i) a history of vascular diseases, autoimmune disorders, or active acute or chronic infections; (ii) use of antibiotics in the last 30 days prior to study enrollment; (iii) a history of intracranial or intraspinal tumor; (iv) a history of metabolic myelopathy; or (v) a history of alcohol or drug abuse.

All RRMS patients received 20 mg of GA subcutaneously every day for a period of 2 years. During this period, the patients underwent clinical assessments, and peripheral blood samples were collected during their outpatient visits at intervals of 0, 3, 6, and 12 months. Patients with symptoms suggestive of a clinical relapse were treated at the University of Maryland Multiple Sclerosis Center. Clinical relapse was defined as a significant deterioration in existing symptoms or the emergence of new neurological deficits, without the presence of fever or active infections and lasting for more than 24 hours. All patients also underwent an EDSS evaluation during each visit. A neurologist thoroughly examined consultation records, clinical records, and inpatient records to verify the completeness of the data obtained. Patients undergoing a relapse were given 1 g of Solu-Medrol i.v. for 3 days, and a Prednisone taper was also used after the Solu-Medrol in certain patients. Blood samples were obtained prior to Solu-Medrol treatment in these patients. Response to therapy was defined as absence of clinical relapse, absence of sustained EDSS worsening, and no new/enlarging T2 or gadolinium-enhancing lesions on MRI during the 2-year time span after the initiation of GA therapy (18). According to these criteria, the present cohort consisted of 11 responders (mean age: 43; range 27–60 years; 55% of them female) and 4 non-responders (mean age: 31; range 22–36 years; 75% female). All non-responder patients enrolled in the study were treatment naïve. The present study was approved by the Institutional Review Board of University of Maryland Baltimore.

### Collection of PBMC, total RNA purification, and cDNA synthesis

We collected PBMC from each patient by using BD Vacutainer CPT tubes (Becton Dickinson, Franklin Lakes, NJ). The mononuclear cells, comprising several populations of immune cells, including monocytes, T and B lymphocytes, dendritic cells, and natural killer cells, were isolated from fresh blood as previously described (15). RNA isolation was performed the same day. RNA purification was performed with a RNeasy Mini Kit (Qiagen, Santa Clarita, CA) according to the manufacturer’s instructions, as described elsewhere (15).

### Real-time quantitative PCR

Real-time PCR was performed using a StepOne Real-Time PCR system (Applied Biosystems, Foster City, CA). The primers for the human RGC-32, FasL, SIRT1, and IL-21 genes were designed and synthesized by Integrated DNA Technologies (IDT, Coralville, IA) (**Supplementary Table 1**) and used in conjunction with LightCycler FastStart DNA Master SYBR Green I (Roche), along with sample cDNA, according to the manufacturer’s protocol. The whole protocol for normalized mRNA value (NRV) calculation for each sample was described elsewhere (16).

### Western blot

We performed Western blotting for p-SIRT1 and JNK1 p54 using patients’ PBMC samples that were previously lysed in RIPA buffer and processed for total protein extraction. The whole protocol was previously described elsewhere (25). For p-SIRT1 detection, we used a rabbit monoclonal IgG anti SIRT1 phosphorylated at Ser47 (Cell Signaling Technologies, Danvers, MA); for JNK1, we used a rabbit polyclonal IgG anti-JNK1 (C-17, Santa Cruz Biotechnology, Dallas, TX). In the same samples, we analyzed β-actin using a rabbit polyclonal IgG anti-β-actin (Rockland, Pottstown, PA) as a loading control for sample normalization. The anti-JNK1 antibody recognizes both the p46 and p54 isoforms; however, we focused on the p54 isoform and chose it for our subsequent analysis.

### Statistical analysis

A mixed model for repeated measures, with the MSDA scores as the dependent variable, the time point (month) and clinical status (relapse or stable; responders or non-responders) as independent variables, and patients as a random variable, has been used for evaluating the difference in MSDA scores between relapsing and stable patients, and between non-responders and responders, respectively. Pearson correlation analysis was conducted to examine the association between two numerical variables. For each individual biomarker and for the MSDA scores, we used receiver operating characteristic (ROC) curve analysis to assess predictive accuracy. The predictive probabilities of binary outcomes regarding clinical state and response to GA treatment were reported as a C-statistic or area under the curve (AUC, represented as a percentage, with a perfect score being 100% predictability). Statistical analysis was performed using IBM SPSS Statistics software version 22, SAS Software and GraphPad Prism software version 9. p values <0.05 were considered statistically significant.

## Results

### Selection of cut-off values for all six individual biomarkers

The biomarkers represented here were investigated in our previous studies and are based on the RGC-32 interactome, being major proteins interacting with or being regulated by RGC-32, a molecule which has been shown to play a major role in cell cycle regulation and differentiation, including in cells with critical role in neuroinflammation and MS pathogenesis such as CD4^+^ T cells and astrocytes (**Supplementary Figure 1**) (26, 27). For each of the six biomarkers used for the MSDA index, two cut-off values were obtained by using ROC analysis from our previously published data (15–18), one value for detecting relapse and another for detecting response to GA (**Supplementary Figure 2**).

Thus, for detecting relapse, we used a cut-off value of <1.27 for the RGC-32/L13 ratio (71% sensitivity and 95% specificity), <52.6 for the FasL/L13 ratio (81% sensitivity, 95% specificity), >16.9 for the IL-21/L13 ratio (54% sensitivity, 88% specificity), <3.05 for the SIRT1/L13 ratio (54% sensitivity, 81% specificity), <0.11 for the p-SIRT1/β-actin ratio (60% sensitivity, 72% specificity), and >1.2 for the JNK1 p54/β-actin ratio (56% sensitivity, 80% specificity), respectively (**Supplementary Figure 2**). For detecting non-response to GA treatment, we used the following cut-off values: <2.52 for the RGC-32/L13 ratio (71% sensitivity, 92% specificity), <85.4 for the FasL/L13 ratio (85% sensitivity, 92% specificity), >11.9 for the IL-21/L13 ratio (81% sensitivity, 89% specificity), <4.33 for the SIRT1/L13 ratio (54% sensitivity, 73% specificity), <0.3 for the p-SIRT1/β-actin ratio (64% sensitivity, 63% specificity), and >1.3 for the JNK1 p54/β-actin ratio (66% sensitivity, 91% specificity) (**Supplementary Figure 2**).

### Generation of the MSDA score

The MSDA score was calculated by combining the arbitrary values based on mRNA values for RGC-32, FasL, IL-21, and SIRT1 (measured by real-time PCR and expressed as a ratio to L13) (15, 16) and protein values for p-SIRT1 and JNK1 p54 (measured by Western blotting and expressed as ratios to β-actin) (17, 18).

**Figure 1:**
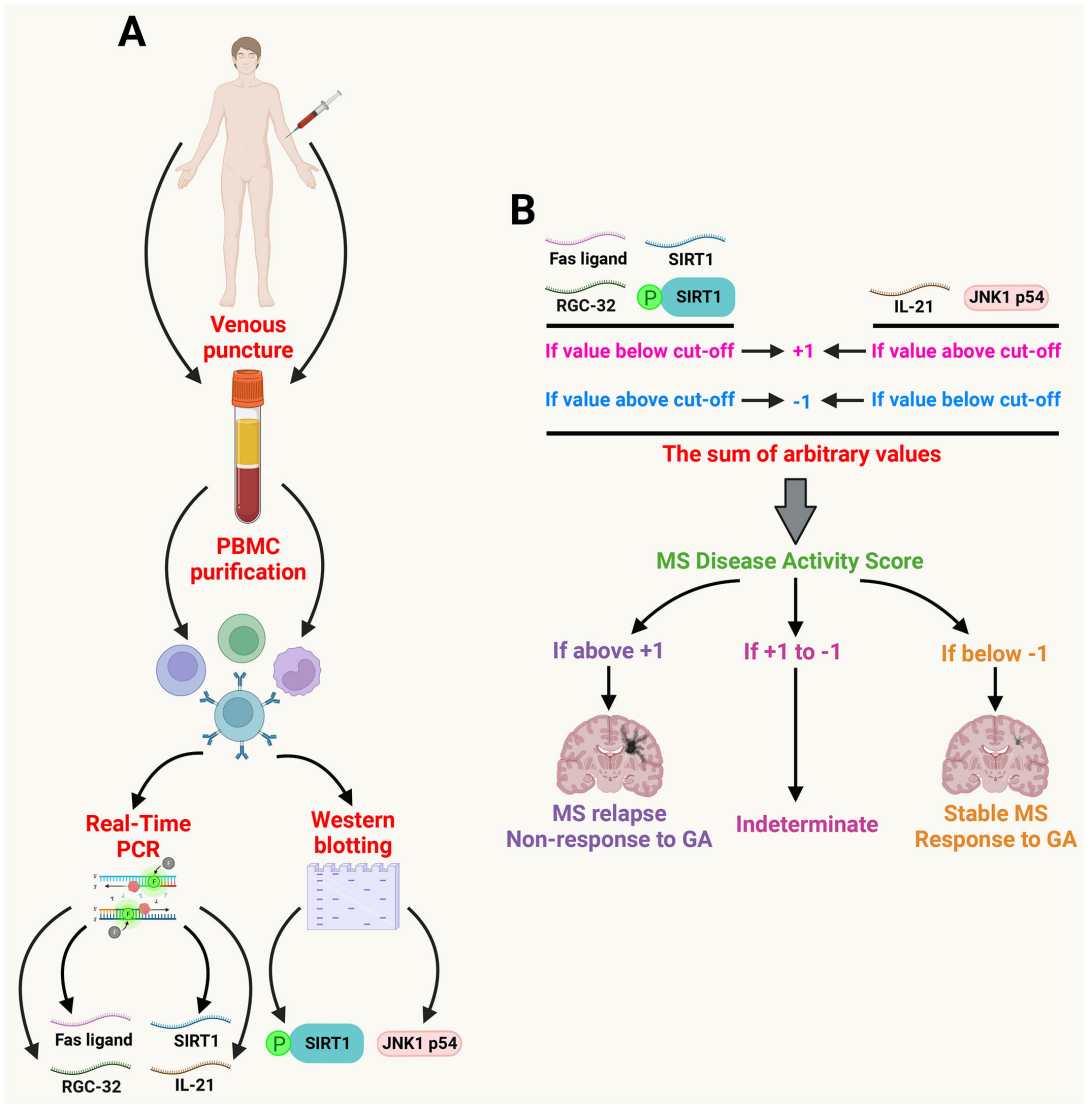
Generation of the MSDA score. A schematic representation of the general methodology used for the generation of MSDA scores. **(A)** the PBMC were first isolated from venous blood of MS patients. The transcript levels of RGC-32, SIRT1, FasL, and IL-21 were determined by Real-Time PCR. The protein levels of phospho-SIRT1 and JNK1 p54 were determined by Western blot. **(B)** The calculated transcript or protein ratios were assigned an arbitrary positive value (+1) or negative value (–1) according to the cut-off for each individual biomarker. These arbitrary values were then summed to generate an MSDA score for disease activity and for response to GA. An MSDA score above +1 indicated a relapse or non-response to GA treatment. An MSDA score less than -1 indicated that a patient was stable. An MSDA score between +1 and -1 was considered indeterminate. (Created with BioRender.com).

First, for each of the biomarkers, we compared the ratios obtained with the above-mentioned cut-off values, allowing us to assign an arbitrary positive or negative value for each biomarker. Any value above the cut-off for RGC-32, FasL, SIRT1, or pSIRT1, or below the cut-off for IL-21 or JNK1 p54 was considered negative, and such biomarkers were assigned an arbitrary -1 value. A -1 value indicated that the patient was either stable or was responding to GA therapy (**Figure 1**). On the other hand, any value below the cut-off for RGC-32, FasL, SIRT1, or p-SIRT1, or above the cut-off for IL-21 or JNK1 p54 was considered positive, and such biomarkers were individually assigned an arbitrary +1 value. A +1 value indicated that the patient had either had a relapse or was not responding to GA therapy (**Figure 1**). Overall, we first assigned the -1 and +1 values for the relapse vs. stable patients in relation to the cut-off values for the clinical stability. Then we assigned another value (-1 and +1) for the responders vs. non-responders in relation to different cut-off values for the response to treatment.

These arbitrary values for individual biomarkers were then summed up to generate an MSDA score indicating disease activity or response to GA therapy, respectively. We used all 6 biomarkers (6-biomarker MSDA) or 4 biomarkers (SIRT1 and pSIRT1 excluded). SIRT1 and pSIRT1 were excluded from the 4-biomarker MSDA score as these 2 biomarkers had the lowest specificity for detecting the 2 outcomes when taken individually, and therefore, we wanted to see whether removing them would improve the overall specificity of the 4-biomarker score vs. the 6-biomarker score.

An MSDA score above +1 indicated that the patient had relapsed or was not responding to treatment. A score below -1 indicated that the patient was stable or was responding well to treatment. An MSDA score between +1 to -1 was considered indeterminate (**Figure 1**). We recommend that patients with an indeterminate score undergo close follow-up and a repeated MSDA score determination after 1-3 months or at any time that the patient displays one or more new symptoms compatible with a relapse or non-response to therapy.

### Application of MSDA score to MS patients

**Figure 2:**
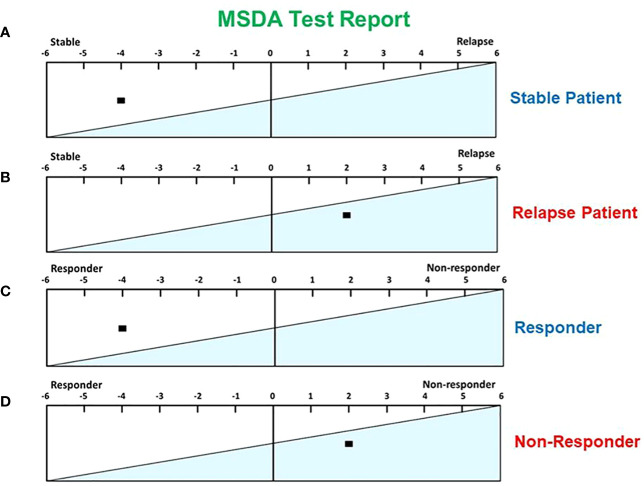
Examples of MSDA scores in different scenarios. **(A)** An MSDA score of -4 was found in a stable MS patient. **(B)** An MSDA score of +2 was found in an MS patient during a relapse. **(C)** An MSDA score of -4 was found in a responder to GA treatment. **(D)** An MSDA score of +2 was found in a patient who did not respond to GA treatment.

In **Figure 2A**, we show an example of a stable patient from our study whose calculated MSDA score was -4. **Figure 2B**, in contrast, is an example of a patient undergoing a relapse, with an MSDA score calculated at +2. Similarly, **Figure 2C** shows an example of a patient with a good response to GA treatment, as defined by our criteria (see above) and an MSDA score of -4, whereas **Figure 2D** depicts results for a non-responder patient, with an MSDA score of +2.

**Figure 3:**
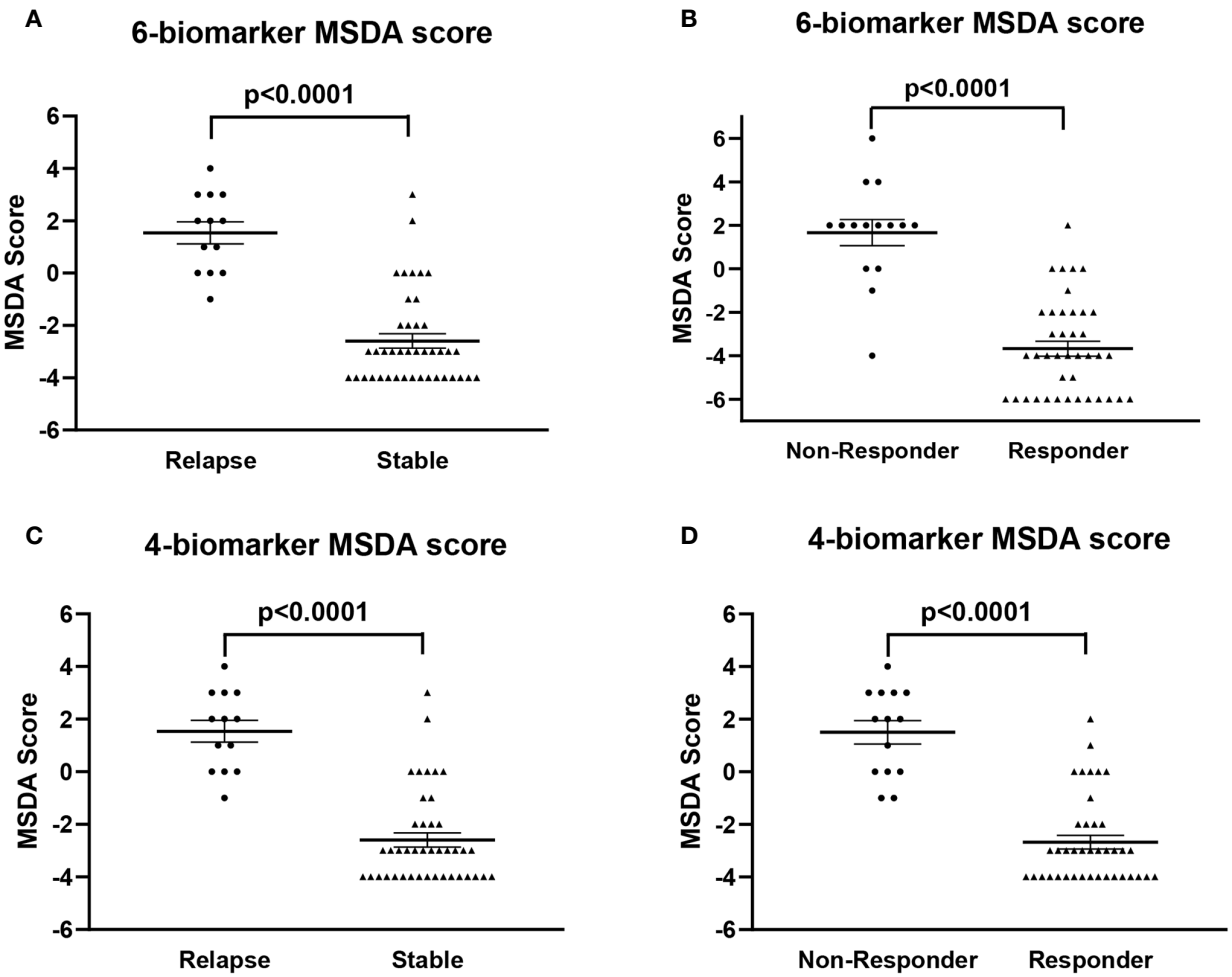
Comparisons of all MSDA scores using six and four biomarkers in the MS patients included in the study. **(A)** Comparison of the MSDA scores obtained with six biomarkers between stable and relapse patients. The relapsing patients had significantly higher MSDA scores than did the stable patients. **(B)** The average of the six-biomarker MSDA scores was also significantly higher in patients who did not respond to GA therapy than in responders. **(C)** The four-biomarker MSDA scores presented significantly higher levels in relapse patients than in those whose disease was stable. **(D)** The average of the four-biomarker MSDA scores was significantly higher in patients who did not respond to GA therapy than in responders. All values are shown as means ± SEM.

Then, we were interested to see whether there was any significant difference in the MSDA scores between stable and relapse patients and between responders and non-responders, respectively. First, we used the mixed model for repeated measures described in the materials and methods for 6 biomarkers. This model indicated significant differences between the relapsed and stable patients (p<0.0001; **Figure 3A**), when there was no difference in the time point (month) (p=0.1252; **Supplementary Table 2**). Similar results were obtained for responders vs. non-responders, where this model indicated a significant difference between the two groups (p<0.0001; **Figure 3B**), when there was no difference in the time point (month) (p=0.435; **Supplementary Table 3**).

Then, we used the same model to compare the scores when using four biomarkers (RGC-32, FasL, Il-21, and JNK1p54). This model indicated significant differences between relapsed and stable patient (p<0.0001; **Figure 3C**) when there was no difference in the time point (month) (p=0.9817; **Supplementary Table 4**). Similar results were obtained for responders vs. non-responders (p<0.0001; **Figure 3D**), with no differences in the time point (p=0.5403; **Supplementary Table 5**).

We then compared the scores for the six- and the four-biomarker MSDAs and we found similar specificity in detecting responses to GA therapy (90%) (**Supplementary Figure 3**). We also found that the six-biomarker MSDA had a slightly better specificity in detecting a relapse (89.5% vs. 84%) (**Supplementary Figure 3**). In terms of sensitivity, we found that the six-biomarker MSDA had a slightly better sensitivity in detecting responses to GA (92% vs. 84%), but a lower sensitivity than the four-biomarker MSDA in detecting relapse (68% vs. 81%) (**Supplementary Figure 3**).

**Figure 4:**
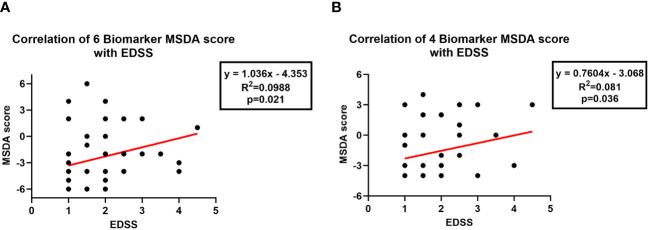
Correlation of MSDA scores with EDSS scores. Correlations between MSDA and EDSS scores were evaluated using Pearson’s correlation. **(A)** The six-biomarker MSDA scores were positively correlated with EDSS scores. **(B)** The four-biomarker MDSA scores were also positively correlated with EDSS scores.

Finally, we were interested in determining how well the MSDA scores correlated with the EDSS results in patients and whether the MSDA scores could be reliably used to predict MS progression. We found that both the six-biomarker (p=0.02) and the four-biomarker MSDA scores (p=0.03) were positively correlated with the EDSS, with higher MSDA scores correlating with higher EDSS scores (**Figure 4A, B**). Although the results reached statistical significance, it should be noted however that the correlation between MSDA scores and EDSS was rather weak, with a Pearson r correlation coefficient of 0.31 for the 6-biomarker score and 0.28 for the 4-biomarker score.

## Discussion

In the present study, we investigated a new multi-biomarker blood test, the MSDA, to measure disease activity and response to treatment in patients with RRMS. We found in preliminary investigation here that this test provided an accurate, simple, and reproducible score on a scale from -6 to +6, based on the blood levels of six biomarkers that reflect various pathophysiologic mechanisms at the level of the peripheral immune system (28). In addition, these markers have been individually evaluated by our team in previous studies (15–18), in terms of their ability to predict relapses and response to GA. The reliability of these individual tests suggested that the use of a combination of such single scores could offer a simple approach for following MS disease activity and progression, as well as for monitoring the benefits of a pharmacological treatment. We have now found that combining the six aforementioned individual test scores (the six-biomarker MSDA) is particularly useful.

Our MSDA scores have proven to be an easy test to use to identify MS relapsing vs. stable patients. In addition, they can be used to guide treatment and to monitor the therapeutic response to GA in RRMS patients, and an analogous MS response-to-therapy score can potentially be established for other available DMT. Moreover, the MSDA test can help MS physicians assess clinically challenging situations, such as when clinical measures are confounded by other inflammatory or non-inflammatory changes from pseudo-relapses in an MS patient (29, 30).

Our results showed that the MSDA with six biomarkers had a sensitivity of 68% and 92% for detecting relapses and response to GA, respectively, and a specificity of 89% and 90% for detecting relapses and response to GA, respectively. For the MSDA with four biomarkers, we found a sensitivity of 81% and 84% for detecting relapses and response to GA, respectively, and a specificity of 84% and 90% for detecting relapses and response to GA, respectively. Except for the sensitivity for detecting relapses, which remained rather low, these values were better than the sensitivities and specificities obtained for the single biomarkers when each was evaluated individually; the sensitivity for detecting response to GA, in particular, was greatly improved. These results suggest that combining the scores for four or six individual biomarkers improved the test’s performance and that both the four- and six biomarker-based MSDA scores can be reliably used to predict disease activity and response to GA. In addition, the MSDA scores were positively correlated with EDSS scores. Although the correlation coefficient was rather weak for each correlation, probably because of the small sample size, these results suggest that our MSDA score may also be predictive of the risk for progression and worsening disability in RRMS patients.

At the present time, there are many molecular biomarkers proposed for MS (e.g., neurofilament light chains, tubulins, and heat shock proteins) (14, 31). Also, thus far, none of the biomarkers has been validated for predicting MS disease evolution or response to treatment (32). Furthermore, the existing clinical and imaging biomarkers do not currently allow for good prognostic prediction (33). Moreover, most of the proposed biomarkers are expensive in terms of cost of analysis (MRI, immunogenic profile), are invasive (lumbar puncture), and are still of uncertain reliability. On the other hand, the proposed MSDA test has only limited invasiveness and a potentially lower cost. Since it combines the levels of six or four biomarkers, as opposed to measuring only one or two inflammatory markers (e.g., the widely used oligoclonal bands or the IgG index in spinal fluid), we believe that the MSDA offers a more accurate assessment of the immunopathological processes underlying relapse. It also has the advantage of using peripheral venous blood and therefore not requiring spinal fluid.

Micro-RNAs regulate gene expression through post-transcriptional modifications. Because of their ability to modulate immune response through gene regulation in key cells involved in the pathogenesis of MS, such as CD4^+^ T cells, micro-RNAs are currently recognized as another class of emerging biomarkers in MS (34, 35). A number of studies have shown that several miRNAs could serve as potential useful biomarkers for MS disease activity and response to treatment. For instance, one study analyzed four different miRNAs in the cerebrospinal fluid of patients with early, highly active RRMS and compared their expression to that in other neurological disorders. The authors found that RRMS patients had significantly higher levels of miR-106a-5p, which was associated with a high number of oligoclonal bands. They also suggested that miR-106a-5p can be considered a biomarker of disease activity (36). Another study showed that a higher expression of miR-648a is directly correlated with more frequent relapses in RRMS patients (37). The expression of miR-155 (which enhances the differentiation of Th17 and Th1 cells) is increased in the serum of MS patients, especially during relapses (38). Expression of miR-320a was found to be decreased in B cells of MS patients and may contribute to increased blood-brain barrier permeability and neurological disability (39). Giuliani et al. have found significantly higher plasma levels of miR-34a and miR-125a-5p, as well as lower miR-146a-5p levels, in RRMS patients than in healthy controls. Moreover, a 4-month treatment with dimethyl fumarate significantly reduced the miR-125a-5p and miR-146a-5p levels (40). Significant differences were also found in miR-548a-3p expression at 6 months after initiation of fingolimod in patients whose MS fulfilled the “no evidence of disease activity” (NEDA)-3 criteria at 2 years. miRNA-548a-3p levels were also higher in these patients than in patients with evidence of disease activity (41). These results are promising, but because of the vast heterogeneity in the patient cohorts examined at present there is no consensus regarding any panel of miRNA-based biomarkers for either disease activity or response to therapy.

An important point to consider is that the MSDA score is designed to be used in individuals who have already been diagnosed with RRMS, and it is not a diagnostic tool for RRMS. It does offer an objective and quantitative score beyond a physical exam that can be used to assess the degree of effectiveness of GA in controlling RRMS. It is also possible that our study here could pave the way for the use of this test to study the efficacy of other widely used DMT in the treatment of RRMS, including beta-interferons, teriflunomide, dimethyl fumarate, sphingosine-1-phosphate receptor modulators, natalizumab, and anti-CD20 monoclonal antibodies.

In summary, our data strongly suggests that MSDA can be used to identify active relapse in RRMS patients and that it has the potential to help clinicians monitor MS patients with high risk of relapse and/or inadequate response to therapy. By predicting a patient’s therapeutic response and the risk of relapse, this test has the potential to complement other measures and optimize clinical decision-making. This combination of parameters seems to represent a very promising and easily obtained composite biomarker for predicting future disease activity. However, it is important to note that our findings must still be confirmed in larger prospective studies involving longer follow-up, to evaluate whether the predictive potential demonstrated in this study on a group level can be confirmed at the level of the individual patient. Nevertheless, the results of this study indicate that MSDA has the potential to become one of the biomarker-based tests of choice for following and monitoring MS patients.

## Data availability statement

The raw data supporting the conclusions of this article will be made available by the authors, without undue reservation.

## Ethics statement

The studies involving humans were approved by Institutional Review Board of University of Maryland Baltimore. The studies were conducted in accordance with the local legislation and institutional requirements. The participants provided their written informed consent to participate in this study.

## Author contributions

AT: Writing – review & editing, Writing – original draft, Validation, Formal Analysis, Conceptualization. FA: Writing – original draft, Validation, Formal Analysis, Conceptualization. DB: Writing – review & editing, Methodology. HC: Writing – original draft, Validation, Formal analysis. AM: Writing – review & editing, Methodology. VN: Writing – review & editing, Methodology. JC: Writing – review & editing, Methodology. VR: Writing – review & editing, Writing – original draft, Validation, Supervision, Conceptualization. HR: Writing – review & editing, Writing – original draft, Validation, Supervision, Conceptualization. HC:
