# Supplementary material for: Multiple sclerosis disease activity, a multi-biomarker score of disease activity and response to treatment in multiple sclerosis
Source: Front Immunol. 2024 Jun 27;15:1338585. doi: 10.3389/fimmu.2024.1338585 (PMC11236682; doi:10.3389/fimmu.2024.1338585)
Supplement: Supplementary file 1 [file DataSheet_1.pdf]

Supplementary Material

1. Tables

Supplementary table 1 | Human primers used for Real Time PCR

Gene symbol	Primers sequence
FasL	For: 5’- GCCCATTTAACAGGCAAGTC -3’<br>Rev: 5’- ATCACAAGGCCACCCTTCTT -3’
RGC-32	For: 5’- AGGAACAGCTTCAGCTTCAG -3’<br>Rev: 5’- GCTAAAGTTTTGTCAAGATCAGCA -3’
SIRT1	For: 5’- TGGCAAAGGAGCAGATTAGTAG -3’<br>Rev: 5’- GGCATGTCCCACTATCACTGT -3’
L13	For: 5’- CGTGCGTCTGAAGCCTACA -3’<br>Rev: 5’- GGAGTCCGTGGGTCTTGAG -3’

Supplementary table 2 | Statistical analysis of relapse vs stable MS patients (6 biomarkers)

**Type 3 Tests of Fixed Effects**				
Effect	Num DF	Den DF	F Value	Pr > F
Month	1	38	2.46	0.1252
Clinical Status	1	38	38.13	<.0001

**Least Squares Means**			
Effect	Clinical Status	Estimate	Standard Error
Clinical Status	Relapse	1.261	0.7162
Clinical Status	Stable	-3.1317	0.4896

DF: degree of freedom; Num DF: Numerator DF; Den DF: Denominator DF

Supplementary table 3 | Statistical analysis of responders vs non-responders MS patients (6 biomarkers)

**Type 3 Tests of Fixed Effects**				
Effect	Num DF	Den DF	F Value	Pr > F
Month	1	39	4.35	0.435
Clinical Status	1	39	26,9.	<.0001

**Least Squares Means**			
Effect	Clinical Status	Estimate	Standard Error
Clinical Status	NR	1.6266	0.847
Clinical Status	R	-3.5184	0.5148

R: Responders to treatment with GA: NR: Non-responders to GA treatment.

Supplementary table 4 | Statistical analysis of relapse vs stable MS patients (4 biomarkers)

**Type 3 Tests of Fixed Effects**				
Effect	Num DF	Den DF	F Value	Pr > F
Month	1	38	0	0.9817
Clinical Status	1	38	23.25	<.0001
**Least Squares Means**				
Effect	Clinical Status	Estimate	Standard Error	
Clinical Status	Relapse	0.7358	0.6078	
Clinical Status	Stable	-2.2153	0.4091	

DF: degree of freedom; Num DF: Numerator DF; Den DF: Denominator DF

Supplementary table 5 | Statistical analysis of responders vs non-responders MS patients (4 biomarkers)

**Type 3 Tests of Fixed Effects**				
Effect	Num DF	Den DF	F Value	Pr > F
Month	1	39	0.38	0.5403
Clinical Status	1	38	20.09	<.0001
**Least Squares Means**				
Effect	Clinical Status	Estimate	Standard Error	
Clinical Status	NR	1.1248	0.6963	
Clinical Status	R	-2.5276	0.4231	

R: Responders to treatment with GA: NR: Non-responders to GA treatment

2. Figures

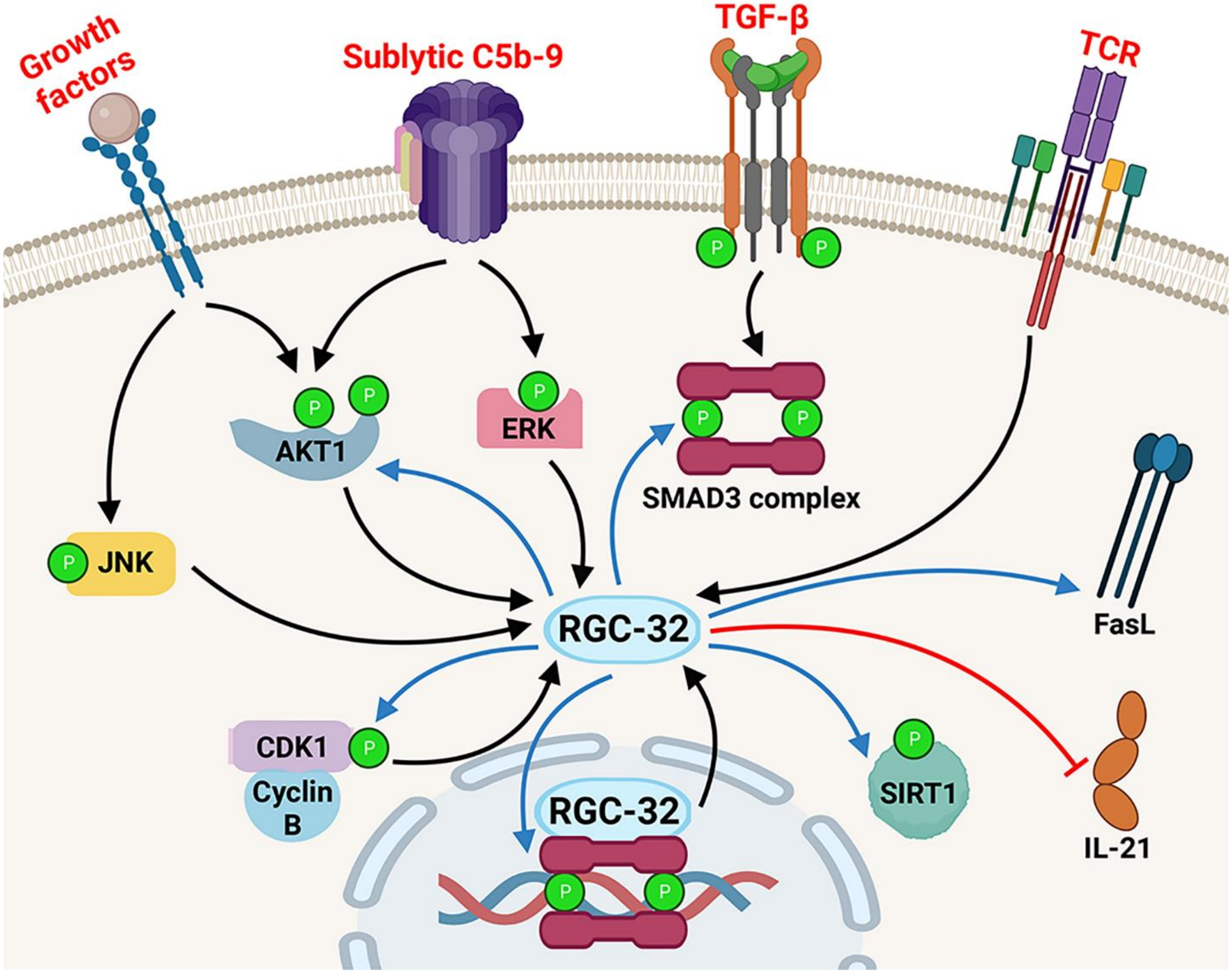

Supplementary Figure 1. RGC-32 interactome

This drawing depicts an overall network of molecular interactions centered around RGC-32. Each of the six biomarkers selected for the generation of the MSDA score was found to interact with RGC-32. For instance, RGC-32 was found to regulate the expression of SIRT1, IL-21 and FasL and JNK1 was found to regulate RGC-32 expression. RGC-32 also regulates other processes, such as SMAD3 nuclear translocation after TGF-β stimulation and cell cycle promotion through the activation of cyclin-dependent kinase 1 (CDK1)/cyclin B complexes and AKT1. (Created with BioRender.com)

	Cut-off values for detecting relapses			Cut-off values for detecting non-response to GA		
Biomarker	Cut-off	Sensitivity (%)	Specificity (%)	Cut-off	Sensitivity (%)	Specificity (%)
RGC-32 mRNA	< 1.27	71	95	< 2.52	71	92
FasL mRNA	< 52.6	81	95	< 85.4	85	92
IL-21 mRNA	> 16.9	54	88	> 11.9	81	89
SIRT1 mRNA	< 3.05	54	81	< 4.33	54	73
pSIRT1 protein	< 0.11	60	72	< 0.3	64	63
JNK1 p54 protein	> 1.2	56	80	> 1.3	66	91

Supplementary Figure 2. Selection of cut-off values for biomarkers used in MSDA score generation

For each individual biomarker used for the MSDA score, a cut-off value was generated by using ROC statistics from our previously published data, one value for detecting relapses and one value for detecting response to GA.

	MSDA score for detecting relapses				MSDA score for detecting response to GA therapy			
	Sensitivity	Specificity	PPV	NPV	Sensitivity	Specificity	PPV	NPV
For 6 biomarkers FasL, RGC-32, IL-21, JNK1 p54, SIRT1, p-SIRT1	0.688	0.895	0.733	0.872	0.923	0.90	0.75	0.973
For 4 biomarkers FasL, RGC-32, IL-21, JNK1 p54	0.813	0.842	0.684	0.914	0.846	0.902	0.733	0.948

Supplementary Figure 3. Predictive accuracies of the six-biomarker vs. the four-biomarker MSDA scores in detecting relapse and response to GA therapy

We found the same specificity for both the six-biomarker and four-biomarker MSDA scores in detecting response to GA, and a slightly better specificity for -biomarker score for detecting relapses. On the other hand, the four-biomarker MSDA score showed a slightly better sensibility in detecting a relapse but a lower sensitivity in detecting response to GA. PPV = positive predictive value, NPV = negative predictive value.
